# Food and Sleep

**Published:** 1890-02

**Authors:** 


FOOD AND SLEEP.—In healthy sleep the person becomes unconscious of the external world, voluntary action ceases, breathing becomes slow, the pulse quiet, and the vessels tend to dilate. This condition of the vessels has been regarded by some as the cause of sleep, rather than its consequence.

We may frequently find that people tend to fall asleep after a very hearty meal. It is possible that this may be partly due to the absorption of certain digestive products, which may act as soporifics, but it is probable that the dilatation of the gastric and intestinal vessels has much to do with the production of sleep, by drawing away blood from the brain.—*Brunton.*

